# A study of primary glomerular diseases in adults; clinical, histopathological and immunofluorescence correlations

**Published:** 2015-10-03

**Authors:** Ananta Satya Narayana Modugumudi, Phaneendra Bobbidi Venkata, Siva Kumar Vishnu Bottla, Radhika Kottu, Rukmangadha Nandyala, Rashmi Patnayak, Amit Kumar Chowhan, Lakshmi Amancharla Yadgiri

**Affiliations:** ^1^Department of Pathology, Sri Venkateswara Institute of Medical Sciences, Tirupati, India; ^2^Department of Nephrology, Sri Venkateswara Institute of Medical Sciences, Tirupati, India; ^3^Department of Radiology, Sri Venkateswara Institute of Medical Sciences, Tirupati, India

**Keywords:** Glomerulonephritis, Immunofluorescence, Renal failure, Membranous nephropathy, Minimal change disease

## Abstract

**Introduction:** The frequency of primary glomerular diseases is variable from one part of the world to the other. Data published from India has shown wide range of variation in the different regions of the country.

**Objectives:** This study reports the frequency of primary glomerulonephritis (GN) in adults in the Rayalaseema region of south India.

**Materials and Methods:** The study is based on prospective evaluation of renal biopsies done during 2 years 4 months period (May 2010-August 2012). A total of 137 cases of primary GN were studied by light microscopy and immunofluorescence (IF). The patients age range between 15-74 years.

**Results:** Most frequent primary GN was membranous nephropathy (MN) constituting 35.8%, followed by minimal change disease (MCD) at 16.7%.

**Conclusion:** This study demonstrates that MN is the most common primary GN encountered in the adults, the second more frequent is MCD. This result is in contrast to previous reports from India where IgA nephropathy (IgAN) and MCD were reported as the most common primary GN in whole population.

**Implication for health policy/practice/research/medical education:**

To determine the frequency of primary glomerulonephritis (GN) in adults in the Rayalaseema region of South India, we conducted a prospective study in 137 cases of primary GN diagnosed by light microscopy and immunofluorescence (IF) on renal biopsies. We found nephrotic syndrome (NS) was the most common clinical presentation followed by rapidly progressive renal failure (RPRF) and membranous nephropathy (MN) was the most common primary renal disease followed by minimal change disease (MCD), which was in contrast to previous reports from India where IgA nephropathy (IgAN) and MCD were reported as the most common primary glomerulonephritis (PGN) in whole population. There is a need for the establishment of the registry on glomerular diseases.

## Introduction

Glomerular disorders constitute one of the major causes of morbidity and mortality (1). Immune mechanisms are responsible for glomerular injury in most cases of primary glomerulonephritis (GN) and many of the secondary GN (2). Immunofluorescence (IF) microscopy enabled us to delineate the different immunoglobulin deposition in the glomerular basement membrane, capillary wall and mesangium which adds to the light microscopic findings (3). Correct diagnosis of GN requires correlation of clinical, biochemical, serological parameters and histopathological examination of renal biopsy by light microscopy, IF and sometimes electron microscopic examination (4).

Renal biopsy data analysis is essential to study the prevalence of biopsy-proven renal disease (BPRD) and its variation and distribution as per geographic areas, socioeconomic conditions, race, age and gender. It also improves the understanding of the utility of renal biopsy and acts as a framework for future research into renal parenchymal disease. In the absence of a renal biopsy registry, there is a paucity of data on the renal disease pattern seen in India.

## Objectives

To study the spectrum of primary glomerular diseases in adults.

## Materials and Methods

It is a prospective study of primary glomerular diseases in native kidneys for which biopsies were performed during the period of May 2010 to August 2012 evaluated at Department of Pathology, Sri Venkateswara Institute of Medical Sciences, Tirupati, including the available clinical, laboratory, histopathological and IF parameters (Tables 1-4).

**Table 1:** Clinical and biochemical parameters noted in the patients (n=137) at the time of renal biopsy (%)

**Edema**	**Hypertension (BP >140/90)**	**Hematuria (macroscopic)**	**Urine examination** **(24 hr. proteinuria)**	**Hyperlipidaemia (Cholesterol >200 mg/dl)**	**Renal impairment (Creatinine >1.5 mg/dl)**
136 (99.2%)	57 (41.6%)	9 (6.6%)	Nephrotic- 60 (43.8), nephritic -46 (33.6%), nephritic-nephrotic-27 (19.7%), normal-4 (2.9%)	86 (62.8%)	69 (50.4%)

(Nephritic->6-10RBC (plenty) +protein< 2+, Nephrotic-no/<6 RBC+protein 3+ or above; Nephritic-nephrotic-RBC >6-10+protein 3+or protein 3+or above; normal-protein <500 mg and RBC <1HPF).

**Table 2:** Glomerular syndromes noted in the patients (n=137) at the time of renal biopsy

**NS**	**RPRF**	**ANIS**	**CRF**	**Isolated proteinuria**
**90 (65.6%)**	**33 (24%)**	**9 (6.6%)**	**4 (2.9%)**	**1 (0.7%)**

Abbreviations: NS, nephrotic syndrome; RPRF, rapidly progressive renal failure; ANIS, acute nephritic syndrome; CRF, chronic renal failure.

**Table 3:** Clinicopathologic correlation of primary glomerular diseases in adults

**Primary glomerular diseases**	**Mean age of patients ± SD (y)**	**M:F ratio**	**Clinical syndromes**	**24 h proteinuria (Nephrotic, nephrotic-nephritic, nephritic)**	**Hypertension (BP> 140/90 mm Hg)**	**Renal impairment (S. creatinine->1.5 mg/dl)**	**Hematuria**
MN (n=49)	(38.16±13.88)	1.7:1	NS -42 (85.7%) ANIS-1 (2.0%) RPGN -6 (12.2%)	Nephrotic -32 (65.3%), nephritic- 10 (20.4%), nephritic –nephrotic-7 (14.3%)	15 (30.6%)	13 (26.5%)	-
MCD (n=23)	(24.47±11.23)	4:1	NS -23 (100%)	Nephrotic-18 (78.2%), nephritic-3 (13%), nephritic –nephrotic-1 (4.3%), normal-1 (4.3%)	0	3 (20%)	-
MPGN (n=23)	(43.26±12.45)	2.2:1	NS -10 (43.5%) RPGN -10 (43.5%) ANIS-2 (8.7%) IP: 1 (4.3%)	Nephritic-14 (60.9%), nephritic –nephrotic-6 (26.1%), nephrotic-2 (8.7%), normal-1 (4.3%)	14 (60.9%)	20 (87%)	2 (8.6%)
RPGN (n=11)	(37±18.4)	2.6:1	RPRF-9 (81.8%) NS-2 (18.2%)	Nephritic –nephrotic-5 (45.5%), nephritic -5 (45.5%), nephrotic- 1 (9.1%)	9 (81.8%)	10 (90.9%)	-
FSGS (n=9)	(40.0±18.55)	1.2:1	NS-9 (100%)	Nephrotic-6(66.7%), nephritic-1(11.1%), nephritic –nephrotic-1 (11.1%), normal-1 (11.1%)	3 (33.3%)	3 (33.3%)	-
CGN (n=9)	(36.33±12)	0.8:1	RPRF-5 (55.6%) CRF-4(44.4%)	Nephritic-5 (55.5%), nephrotic-3 (33.3%), normal-1 (11.1).	9 (100%)	9 (100%)	
PIGN (n=7)	(45.33±15.9)	1.3:1	ANIS-4 (57.1%) RPGN -2 (28.6%) NS-1 (14.3%)	Nephritic –nephrotic -4(57.1%), Nephritic -2 (28.6%), nephrotic- 1 (14.3%)	5 (71.4%)	5 (71.4%)	4 (57.1%)
IgAN (n=3)	(34.33±19)	-	ANIS-2 (66.7%) RPRF-1 (33.3%)	Nephritic-3 (100%)	2 (66.7%)	3 (100%)	2(66.6%)
Mes. PGN (n=3)	(41.55±12.4 )	2:1	NS -3 (66.6%)	Nephrotic-2 (66.6%), nephritic –nephrotic-1 (33.3%)	0	3 (100%)	-

Abbreviations: MN, membranous nephropathy; MCD, minimal change disease; MPGN, membranoproliferative glomerulonephritis; RPGN, rapidly progressive glomerulonephritis; FSGS, focal segmental glomerulosclerosis; CGN, chronic glomerulonephritis; PIGN, post infectious glomerulonephritis; IgAN, IgA nephropathy; Mes.PGN, Mesangial proliferative glomerulonephritis.

**Table 4:** IF findings in patients with each primary glomerular diseases (n = 137)

**Histopathological diagnosis**	**IgG**	**IgA**	**IgM**	**C3**	**C1q**
MN (n = 49)	45 (91.8%)	8 (16.3%)	24 (48.9%)	31 (63.2%)	1 (2.0%)
MCD (n = 23)	0	0	4 (26.7%)	0	0
MPGN (n = 23)	14 (60.8%)	1 (4.3%)	8 (34.7%)	17 (73.9%)	2 (8.69%)
RPGN (n = 11)	5 (45.4%)	5 (45.4%)	6 (54.5%)	8 (72.7%)	1 (9%)
FSGS (n = 9)	0	0	5 (55.6%)	0	0
CGN (n = 9)	7 (77.7%)	2 (22.2%)	3 (33.3%)	4 (44.4%)	0
PIGN (n = 7)	7 (100%)	3 (42.8%)	0	6 (85.7%)	0
IgAN (n = 3)	2 (66.6%)	3(100%)	1 (33.3%)	2 (66.6%)	0
Mes.PGN (n = 3)	0	0	3(100%)	0	0

Abbreviations: IF, immunofluorescence; MN, membranous nephropathy; MCD, minimal change disease; MPGN, membranoproliferative glomerulonephritis; RPGN, rapidly progressive glomerulonephritis; FSGS, focal segmental glomerulosclerosis; CGN, chronic glomerulonephritis; PIGN, post infectious glomerulonephritis; IgAN, IgA nephropathy; Mes.PGN, Mesangial proliferative glomerulonephritis.

### Inclusion criteria

Patients presenting with glomerular diseases to the nephrology department in whom renal biopsy was performed were included in the study.

### Exclusion criteria

The exclusion criteria were presence of non-glomerular diseases, presence of secondary glomerular diseases, children <15 years of age and inadequate biopsies.

Data for each case included were age, gender, history, clinical examination findings including history of diabetes mellitus and hypertension (blood pressure >140/90 mm Hg), laboratory and radiological findings. Laboratory findings included were complete blood count (CBC), complete urine analysis and microscopy, 24 hours urine protein excretion (g/day), serum total protein/albumin, creatinine clearance (ml/min), serum creatinine, serum urea, blood glucose levels, serum lipid profile, serum electrolytes (Na^+^, K^+^, Ca^2+^, P), serological markers of hepatitis B, C and antiretroviral, complement levels (C3 and C4) and antistreptolysin (ASO) titre. Additionally when needed, special tests such as antinuclear antibodies (ANA), Anti-ds DNA, ANCA (p and C) were conducted. Ultrasonography was performed to study the size, morphological features and to exclude any obstruction of the kidney.

Based on the available clinical, laboratory and sonography findings, the main clinical syndromes observed in patients at the time of renal biopsy were noted as:

1. Nephrotic syndrome (NS) was defined as proteinuria ≥3.5 g/day with or without hypoalbuminemia, hyperlipidaemia or lipiduria.
2. Acute nephritic syndrome (ANIS) was defined as haematuria, red blood cell (RBC) casts, azotemia, oliguria, edema, hypertension and proteinuria (<3.5 g/day), which persisted <3 months.
3. Rapidly progressive renal failure (RPRF) was defined as ANIS with acute deteriorated renal function (hours to days) such as a two-fold increase in serum creatinine concentration or a decrease in creatinine clearance by 50%.
4. Chronic renal failure (CRF) was defined as azotemia, proteinuria (1–3.5 g/day) and hematuria that persisted for more than 3 months.
5. Asymptomatic urinary abnormality (AUA) was defined as proteinuria (<1.0 g/day) and hematuria found by routine check-up, without edema, hypertension and abnormal renal function.

Each biopsy was performed with an automated biopsy gun using 18 Gauge needle. Two core biopsies were received, one for light microscopy in 10% buffered formalin and other for IF in normal saline.

The tissue obtained for light microscopy was fixed in 10% buffered formalin, embedded in paraffin, sectioned at 3-4 µm thickness and stained with Haematoxylin & Eosin (H & E). For histochemistry, special stains such as Periodic acid–Schiff (PAS), methenamine silver (Jones) stains were used to delineate the architecture of glomerular basement membrane and martius scarlet blue (MSB) stain was used to detect deposition of fibrin and thrombi.

The tissue obtained for direct IF (DIF) study, preserved in normal saline, was immediately processed for frozen section. Sections were cut in a -20°C cooled chamber of cryostat (Leica CM 1100, Heidelberger Strasse, Nussloch, Germany) at a thickness of 4-5 μm and the sections were air dried and stained with fluorescein isothiocyanate (FITC) conjugated polyclonal rabbit antisera for IgG, IgA, IgM, C3 and C1q separately. The stained sections were examined under an IF microscope (Carl Zeiss Axioster plus HBO 50 fluorescent lamp, Gottingen, Germany) and the location, pattern and intensity of fluorescence were noted.

### Ethical issues

The research followed the tenets of the Declaration of Helsinki. Informed consent was obtained, and the research was approved by the ethical committee of Sri Venkateswara Institute of Medical Sciences (IEC/2011/IEC-177).

### Statistical analysis

Each case data were stored on a database file (Windows Excel).The observations were subjected to statistical analysis by SPSS software. Simple descriptive statistics were used. The continuous variables were expressed in mean with standard deviation and the categorical variables were expressed as proportions.

## Results

Total number of native renal biopsies received during the study period (May 2010-August 2012) was 181. Primary glomerular disease patients constitute 137 (75.6%) cases; secondary glomerular disease patients constitute 41 (22.6%) cases, and 3 inadequate biopsies (1.65%). Patients with secondary glomerular diseases and inadequate renal biopsies were excluded from the study (n=44). Among them 89 (65%) were males and 48 (35%) were females. M: F ratio was 1.85:1.The patients age range from 15 to 74 years. The mean age of patients (n=137) were 37.99 ± 14.74 years and the median age of patients (n=137) were 37.0 (interquartile range: 26.5-49.0).

**Figure 1:**
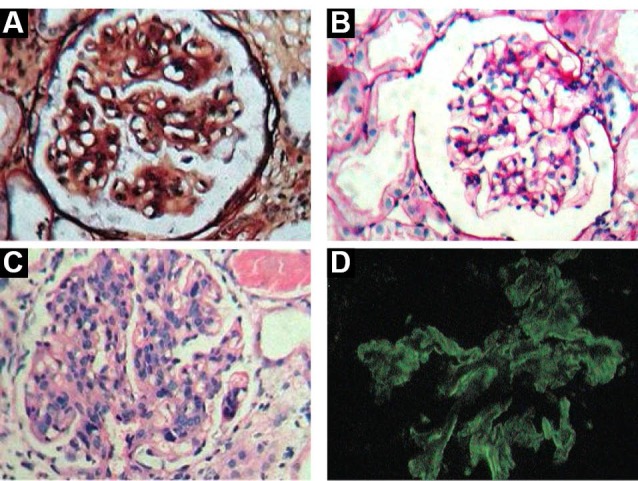
(A) Photomicrograph of MN (JMS×400); glomerulus exhibiting membranous thickening with patent capillary lumina. (B) Photomicrograph of MCD (PAS×400); near normal glomerulus with patent capillary lumina. (C) Photomicrograph of MPGN (H&E×400); glomerulus exhibiting membranoproliferative changes with lobular accentuation and obliterated capillary lumina. (D) Immunofluorescence photomicrograph of MPGN (C3×400) - showing broad interrupted granular peripheral intense staining for C3 along glomerular capillary loops and mesangium.

MN (35.8%) (Figure 1A) was the most common primary glomerular disease encountered commonly in males with a M: F ratio of 1.7:1, majority of them were presented with NS (85.7%) with nephrotic range proteinuria (65.3%), hypertension (30.6%) and minimal renal impairment (26.5%). MCD (16.7%) (Figure 1B) was second most common disease encountered commonly in males with a M: F ratio of 4:1, all presented with NS (100%) with very little renal impairment (20%).

**Figure 2:**
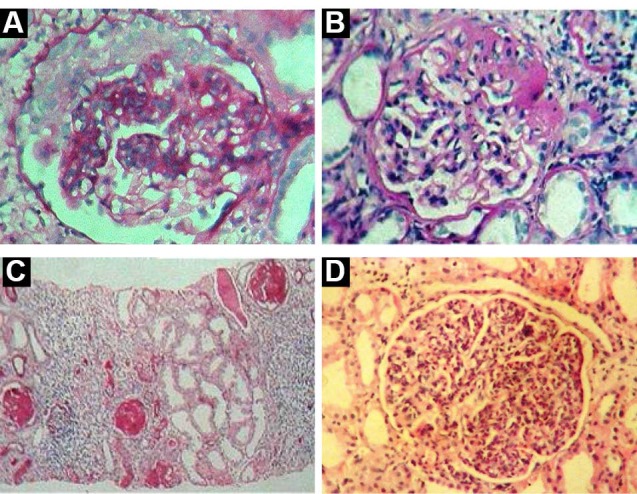
(A) Photomicrograph of RPGN (PAS×400); glomerulus displaying hemi circumferential cellular crescent in the bowman’s space with compressed glomerular loops. (B) Photomicrograph of FSGS (PAS×400);glomeruli exhibiting focal and segmental sclerosis with segmental obliteration of capillary lumen at sclerosed area with synechiae formation. (C) Photomicrograph of CGN (PAS×400); globally sclerotic (obsolescent) glomeruli, markedly atrophic tubules with thick basement membrane, interstitial fibrosis and hyperplastic arteriosclerosis. (D) Photomicrograph of PIGN (H&E×400); glomerulus displaying global intracapillary hyper cellularity with enlarged capillary tufts and reduced capillary lumina which are filled with plenty of neutrophils.

MPGN (16.7%) (Figure 1C and 1D) was next most common disease encountered commonly in males with a M: F ratio of 2.2:1, majority presented with NS (43.5%) and (43.5%) with renal impairment (87%) and hypertension (60.9%). RPGN (8.0%) (Figure 2A) encountered commonly in males with a M: F ratio of 2.6:1, majority presented RPRF (81.8%) with renal impairment (90.9%) and hypertension (81.8%). FSGS (6.6%) (Figure 2B) commonly seen in males with a M: F ratio of 1.2:1, all presented with NS (100%), minimal renal impairment (33.3%) and hypertension (33.3%). CGN (6.6%) (Figure 2C) encountered commonly in females with a M: F ratio of 0.8:1, majority presented RPRF (55.6%) and CRF (44.4%) with renal impairment (100%) and hypertension (100%).

**Figure 3:**
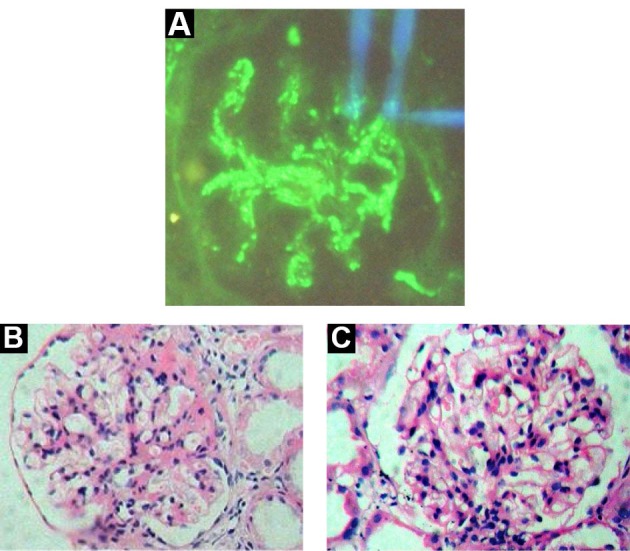
(A) Immunofluorescence photomicrograph of PIGN (IgG×400) -Showing coarse granular positivity for IgG along the capillary basement membrane. (B) Photomicrograph of IgAN (H&E×400); glomerulus displaying global mesangial cellular proliferation with increase in mesangial matrix and segmental sclerosis with synechiae formation. (C) Photomicrograph of Mes.PGN (H&E×400); glomerulus exhibiting diffuse mesangial cellular proliferation (4 or more mesangial cells) with patent capillary lumina.

PIGN (5.1%) (Figures 2D and 3A) encountered commonly in males with an M: F ratio of 1.3:1, majority presented ANIS (57.1%) and RPRF (28.6%) with renal impairment (71.4%), hypertension (71.4%) and haematuria (57.1%). IgAN (2.2%) (Figure 3B) was encountered only in males, presented with ANIS (66.7%) and RPRF (33.3%) with renal impairment (100%), hypertension (66.7%) and haematuria (66.6%). Mes.PGN (2.1%) (Figure 3C) commonly encountered in males with a M: F ratio of 2:1, all presented with NS (100%) with nephrotic (66.6%), and nephritic- nephrotic range proteinuria (33.3%) and renal impairment (100%).

## Discussion

In the current study, we analysed the clinical, laboratory and pathological data of 137 cases of primary glomerular diseases, diagnosed between May 2010 and August 2012 in a single centre in Rayalaseema region. This report is the first systematic review of histological data in our centre. Males outnumbered females in all primary glomerular diseases. This is similar to other epidemiological studies done in India, (5-7) and other countries, (8-11) as shown in Table 5.

**Table 5:** Comparisons of some basic data and common diseases in our series with other published studies

**Variables**	**Present study**	**Das et al(2)(NIMS)**	**Balakrishnan et al (6) (CMC)**	**Chugh and Shakhuja (7) (PGI)**	**Mubarak et al (22) (Pakistan)**	**Li and Liu (16) (China)**	**Chang et al (17)(Korea)**	**Research Group (12)(Japan)**	**Yahya et al (19)(UAE )**
Duration	2010-2012	1990-2008	1986-2002	-	1995-2008	1979-2000	1987-2006	1985-1993	1978-1996
Total no.	137	1849	5415	2947	1793	10002	1818	1850	490
M:F	1.85:1	1.5:1	-	-	1.6:1	1:3	1.02:1	-	-
Mean age	37.9 ± 14	32.27±18.4	-	-	32.9±12.8	31.4±13	-	-	-
NS	65.6	49	65.7	-	49.9	-	-	-	54
ANIS	6.6	9	15.7	-	4.6	-	-	-	-
RPRF	24.8	12	3.4	-	-	-	-	-	-
CRF	2.9	13.6	10.2	-	15.7	-	-	-	12.7
AUA	0.7	9	1.7	-	1.1	-	-	-	29.7
MN	35.8	10	9.5	10	17.2	9.89	12.3	10.6	20.1
MCD	16.7	21.8	10.8	23	5.8	0.93	15.5	17.5	18.3
MPGN	16.7	5.7	2.9	18	1.1	3.38	4	7.5	-
Mes PGN	2.1	7.5	7.3	3	1.9	25.62	-	-	-
RPGN	8	6.5	-	5	3.9	1.9	-	0.9	-
FSGS	6.6	15.3	16.8	9	21.2	6	5.6	4.6	18.3
CGN	6.6	9.7	-	7	11.6	-	-	-	-
PIGN	5.1	8.1	13.5	-	-	2.75	-	-	-
IgA N	2.2	4.4	8.4	4	1.5	40	28.3	47.4	6.3

The most common indication for renal biopsy was NS (65.6% in the whole group), followed by RPRF (24.8%), ANIS (6.6%), CRF (2.9%) and isolated proteinuria (0.7%). These data agree with other studies in which NS is the most frequent indication of renal biopsy (9-13). Conversely in the Italian (8) and Czech (14) registry, AUA are more common than NS, perhaps expressing a tendency to biopsy asymptomatic haematuria or proteinuria.

The most frequently diagnosed lesion in our patients with primary GN was MN (35.8%) followed by MCD (16.7%) and MPGN (16.7%). These three entities comprised 69.2% of primary GN. MN used to be the most common cause of NS in adults and is still cited as the most common cause of NS in adults in widely used renal pathology textbooks. In European studies, it is still common and, notably, more common than FSGS (9).

IgAN is the most common glomerular disease worldwide, but its detection rate varies widely, mostly depending on biopsy indications and mass urinary screening for AUA (15). The low prevalence of IgAN in our series (2.2%) is most probably due to very low rate of renal biopsies in patients with AUA.

The prevalence of IgAN seems to be less in our study while literature review suggests that IgA nephropathy is the commonest primary GN in Asia (12,16,17). We cannot attribute our findings to any sampling/selection error as our work is a prospective study which included all the clinical renal syndromes extending from asymptomatic haematuria/proteinuria to CRF passing in between through acute nephritis, RPRF and NS.

Many patients with IgAN, especially those with asymptomatic haematuria and/or proteinuria, are detected on routine urine screening. Prevalence may therefore appear to be higher in countries with an active urine testing programme and a low threshold for the performance of renal biopsy in patients with isolated asymptomatic hematuria, such as Japan, where testing is routinely performed in schools and in the workplace (12).

The MCD is the next most frequent primary glomerular disease (GD) in this study. The occurrence of MCD (27.3%) in the adult population of our area is almost akin to data from other parts of India (5,6), Korea, (17) Africa (18), UAE (19) and USA (20).

FSGS was not rare among our group and represents 6.6% of primary GD where as other studies from India (6,21), USA (20), Pakistan (22), and Brazil (23) reported FSGS as the most common primary glomerular disease.

In view of that fact of poor socioeconomical conditions, the prevalence of MPGN was 16.7% of primary GD in our study and it is the most common primary glomerular disease reported in Romania (24).

Immunoglobulin M nephropathy (IgMN) has not been observed in our study. A very high prevalence of IgMN has been reported in Thailand, where it constituted the most common renal pathology in adults, seen in 45% of overall cases (25).

With improvement in standards of living, better public health and the early effective antibiotic treatment of pharyngeal infections, it has been reported that the proportion of MPGN and PIGN declined in many countries other than India (5,6) and China (16).

In our study, MCD, FSGS and MN are the most common causes of NS similar to those of other studies done in India (5,6), Italy (8), Spain (10), Japan (12) and Korea (17). IgAN and PIGN are the leading causes of ANIS and haematuria similar to that of study done in Spain (10).

A comparison of the basic data and some common diseases in our series with those of other published studies from the same region and western countries is provided in Table 5. There are several biases regarding demographical, geographical and racial characteristics, differences in indications for renal biopsy, the analyzed clinical syndromes and variations in pathological classification.

It is obvious from the Table 5 that the distribution pattern of primary glomerular diseases in our study did not correspond to other European and some Asian series. Most of these are multicentre studies.

Our present data showed that the pattern of glomerular disease in a referral tertiary care teaching hospital in Rayalaseema region differs from that of other studies done in India and the western world. This difference may be possibly due to the difference in socio-economic status and genetic and environmental factors. Although the result is quite significant in comparison with other studies, further large-scale multi-centre studies should be carried out for a longer period and a national registry for GN should be established to determine current status, better planning, and management of glomerular diseases. This is an endeavour to highlight, at least in part, the details of the primary glomerular diseases occurring in the Rayalaseema region of South India for better clarity and understanding of the prevailing problem.

## Conclusion

NS was the most common clinical presentation in our study followed by RPRF. MN is the most common primary glomerular disease in adults followed by MCD and MPGN. IgAN is found to be unusually low in its incidence despite the absence of selection bias. There is a need for the establishment of the registry on glomerular diseases.

## Limitations of the study

Small sample size and exclusion of paediatric group of population.

## Acknowledgments

The authors wish to acknowledge senior technicians Mrs. Ushanandini and Mr.Venkataramana for the excellent special stains and IF staining.

## Authors’ contribution

M.Ananta Satya Narayana: Data gathering, data interpretation, and manuscript preparation (Thesis work). B.V.Phaneendra: Study design, interpretation of renal biopsies. V.Siva Kumar: Study design and patients support. K.Radhika, Dr.N.Rukmangadha, Dr.Rashmi Patnayak: Interpretation of renal biopsies. Amit Kumar Chowhan: Interpretation of renal biopsies, manuscript edition, and final revision. A.Y.Lakshmi: Ultrasound guidance for renal biopsies.

## Conflicts of interest

The authors declared no competing interests.

## Ethical considerations

Ethical issues (including plagiarism, data fabrication, double publication) have been completely observed by the authors.

## Funding/Support

This research project was approved by Institutional Thesis Approval Committee and the number of this project is 177. This project started at May 28, 2010 and finished at August, 2, 2012.
